# Peripheral memory and naïve T cells in non-small cell lung cancer patients with lung metastases undergoing stereotactic body radiotherapy: predictors of early tumor response

**DOI:** 10.1186/s12935-019-0839-5

**Published:** 2019-05-07

**Authors:** Chao Liu, Qinyong Hu, Bin Xu, Xiaoyu Hu, Huichao Su, Qian Li, Xiaoling Zhang, Jinbo Yue, Jinming Yu

**Affiliations:** 1 Department of Oncology, Renmin Hospital of Wuhan University, Wuhan, 430060 China; 2 Department of Radiation Oncology, Shandong Cancer Hospital and Institute, Shandong Cancer Hospital Affiliated to Shandong University, Shandong Academy of Medical Sciences, Jinan, 250117 Shandong China; 3 Department of Radiation Oncology, Affiliated Hospital of Academy of Military Medical Sciences, Beijing, 100071 China; 4 Department of Gynecologic Oncology, Shandong Cancer Hospital and Institute, Shandong Cancer Hospital Affiliated to Shandong University, Shandong Academy of Medical Sciences, Jinan, 250117 Shandong China

**Keywords:** Memory T, Naïve T, Lung metastases, SBRT, Predictive value

## Abstract

**Background:**

Further analysis of phase I trial of the KEYNOTE-001 has shown that previous radiotherapy improves the outcomes of patients with advanced non-small cell lung cancer (NSCLC) who received pembrolizumab treatment, possibly explained by the radiation-induced specific anti-cancer immunity with a memory effect. In this study, we aimed to investigate the peripheral memory and naïve T cells as predictors of early response in lung metastases post-stereotactic body radiotherapy (SBRT).

**Methods:**

Sixty-six lung metastases patients with NSCLC who received SBRT were enrolled in this study. Analyses of peripheral memory CD4+ T, memory CD8+ T, naive CD4+ T, and naive CD8+ T in NSCLC patients were performed by flow cytometry. Evaluations of the link between immune cells and early radiation response a month after SBRT were carried out via logistic regression analyses.

**Results:**

Higher levels of memory CD4+ T, memory CD8+ T, and lower levels of naïve CD4+ T, CD4+ naïve/memory ratio, and CD8+ naïve/memory ratio were shown in responders compared with non-responders (all *P* < 0.05). Logistic regression analyses of univariate and multivariate revealed that peripheral memory CD4+ T (OR: 0.14, 95% CI 0.04–0.50, *P* = 0.003; OR: 0.17, 95% CI 0.05–0.66, *P* = 0.010), memory CD8+ T (OR: 0.11, 95% CI 0.01–0.87, *P* = 0.037; OR: 0.11, 95% CI 0.01–0.97, *P* = 0.047), naïve CD4+ T (OR: 16.25, 95% CI 3.17–83.13, *P* = 0.001; OR: 12.67, 95% CI 2.26–71.18, *P* = 0.004) and CD4+ naïve/memory ratio (OR: 11.27, 95% CI 2.67–47.58, *P* = 0.001; OR: 8.50, 95% CI 1.90–38.14, *P* = 0.005) were independent predictors for tumor response to SBRT in the lung metastases of NSCLC patients.

**Conclusions:**

The tumor response of lung metastases a month after SBRT independently correlated with peripheral memory CD4+ T, memory CD8+ T, naïve CD4+ T, and CD4+ naïve/memory ratio. These findings could be helpful in incorporating additional treatments to improve clinical outcomes in the case of poor responders.

**Electronic supplementary material:**

The online version of this article (10.1186/s12935-019-0839-5) contains supplementary material, which is available to authorized users.

## Background

To date, radiotherapy, alone and combined with other therapies, such as chemotherapy, targeted therapies, and immunotherapy, is given as a frontline therapy to nearly 60% of all patients newly diagnosed with cancer [1–4]. Stereotactic body radiotherapy (SBRT), known for its high local control rate and insignificant toxicity, has become the primary technique for treating non-small cell lung cancer (NSCLC), especially in the early stage and the oligometastatic types [5–8]. Radiation’s clinical effectiveness has previously been credited to its DNA damage-inducing capacity, which sometimes causes direct tumor-related cell death [9]. Antitumor immunity mobilization has subsequently become a significant contributing factor to the whole clinical efficiency of tumor radiotherapy [10–13].

The “abscopal effect” is a fascinating but sporadic occurrence prominent with SBRT that describes the extra tumor burden regression in non-irradiated spots post-local radiotherapy [14–16]. This special phenomenon has been explained by SBRT’s activation of the anti-tumor immune response [11, 17–19]. Specifically, SBRT induces immunogenic cell stress or death of cancer cells and eases DCs’ recruitment into the tumor bed, DCs’ washout of tumor antigens, and a peak antigen performance in lymph nodes’ T cells. Eventually, primed and activated T cells exit the lymph nodes, home to irradiated and non-irradiated tumors, and kill tumor cells [9, 20].

Despite its ability, clinical tests with SBRT have rarely yielded abscopal effects in patients with advanced cancer, probably due to the immunosuppressive feature of these patients [20]. Researchers have found that CD8+ T cells are required to trigger SBRT’s healing effects on local tumors, suggesting that host immune status is important for SBRT to take effect in cancer patients [10]. However, to date, we have not found a study that has examined peripheral immune cells for predictive roles in tumor response to SBRT in lung cancer patients. Further analysis of the phase I trial of the KEYNOTE-001 has shown that preceding radiotherapies in patients with advanced NSCLC undergoing pembrolizumab treatment results in more favorable outcomes when compared with pembrolizumab treated patients who underwent no earlier radiotherapy, which could be explained by the radiation-induced specific anti-cancer immunity with a memory effect [21, 22]. Our hypothesis was that memory T and naïve T cells could influence the anti-tumor effect of SBRT. We sought, therefore, to study the predictive values of memory CD8+ T, memory CD4+ T, naïve CD8+ T, and naïve CD4+ T for tumor response to SBRT in patients with NSCLC lung metastases.

## Methods

### Patients

The Ethical Committee of the Affiliated Hospital of the Academy of Military Medical Sciences approved our investigation. All patients presented written informed consents before enrolment. We selected 66 patients with NSCLC lung metastases undergoing SBRT between December 2014 and January 2018. To be selected, patients had to meet the following criteria: a histology examination confirming lung metastases, lung metastases treated with SBRT, performance status ≤ 1, age > 18 years, and definitive treatment for prior NSCLC. The exclusion criteria were as follows: patients concurrently receiving other anti-tumor treatments (chemotherapy, immunotherapy, targeted therapy) within 1 month of SBRT; patients who had received anti-tumor treatment or steroids 3 months prior to enrollment; hematonosis, systemic lupus erythematosus, ulcerative colitis, hyperthyroidism, scleroderma, rheumatoid arthritis, chronic liver disease, renal diseases, and other malignant tumors. The baseline characteristics of patients considered were age, sex, smoking history, performance status, histological types, size of lung metastases, primary tumor (T) stage, node (N) stage, and American Joint Committee on Cancer (AJCC) stage according to AJCC-7 criteria [23].

### Flow cytometry

Additional file 1: Figure S1 shows representative flow cytometry plots and gating. We collected 4 mL of fresh blood from patients 7 days before SBRT. The flow cytometry protocol, the same as described in our previous study [24], was used to detect memory CD8+ T (CD3+ CD8+ CD45RA–CD45RO+), naive CD8+ T (CD3+ CD8+ CD45RA+ CCR7+), memory CD4+ T (CD3+ CD4+ CD45RA–CD45RO+), and naïve CD4+ T (CD3+ CD4+ CD45RA+ CCR7+).

### SBRT and tumor response

SBRT was employed to treat lung metastases by CyberKnife. BED_10_ was calculated using the formula D × [1 + d/(α/β)]; D represents total dose, d stands for dose per fraction, and α/β = 10 [25]. The SBRT dose prescribed for radiation therapy was the responsibility of the oncologist carrying out the radiation treatment and was prescribed in consideration of normal tissue tolerances; our patients were dosed with 5 fractions of 10 Gy or 10 fractions of 7 Gy.

Tumor response was evaluated 1 month after SBRT using a computed tomography (CT) or positron emission tomography-computed tomography (PET-CT) according to the RECIST 1.1 guideline [26]. On the one hand, a minimum 20% increase in the diameter of targeted lung metastases was used to mark a progressive disease (PD), while a minimum 30% reduction in the diameter of targeted lung metastases represented a partial response (PR). On the other hand, the absence of an adequate shrinkage to qualify for PR or an acceptable increase to qualify for PD marked a stable disease (SD), whereas, the diminishing targeted lung metastases represented a complete response (CR).

### Statistical analysis

The link between predictors and early radiation response was assessed by analyses of logistic regression. Univariate analytical predictive findings with *P *< 0.1 were further subjected to multivariate analyses. Comparisons of immune cells differences between responsive and non-responsive patients were carried out using the independent Student t-test. Cut-off values representing immune cells’ ability to discriminate between responsive and non-responsive patients were determined using the receiver operating characteristic (ROC) curve and were used to determine high and low immune cells. Cut-off values for memory CD8+ T, naive CD8+ T, memory CD4+ T, naive CD4+ T, CD4+ naïve/memory ratio, and CD8+ naïve/memory ratio were 43.5, 33.1, 58.9, 20.9, 0.32, and 0.99, respectively. Statistical significance was marked by *P*-value < 0.05. Data were analyzed on the SPSS 23.0 software (SPSS Inc., Chicago, IL).

## Results

### Baseline characteristics

**Fig. 1:**
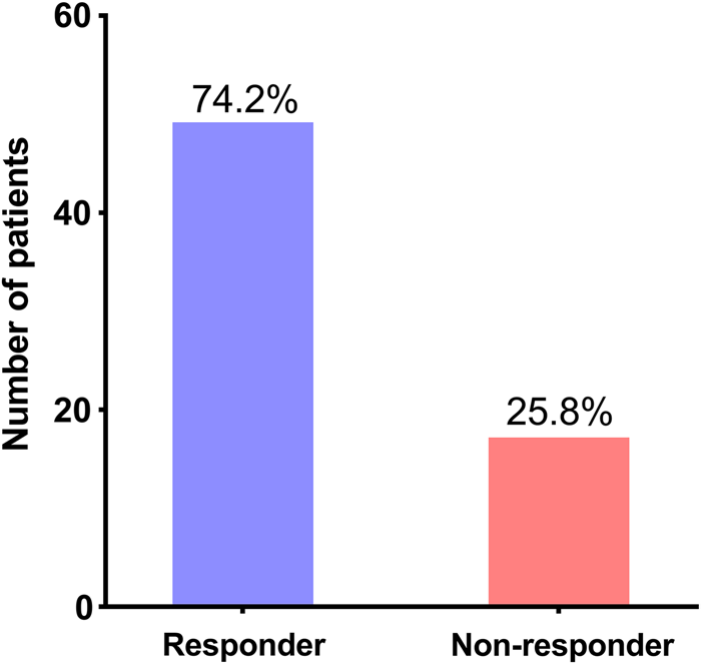
Numbers and proportions of responders and non-responders for SBRT

**Fig. 2:**
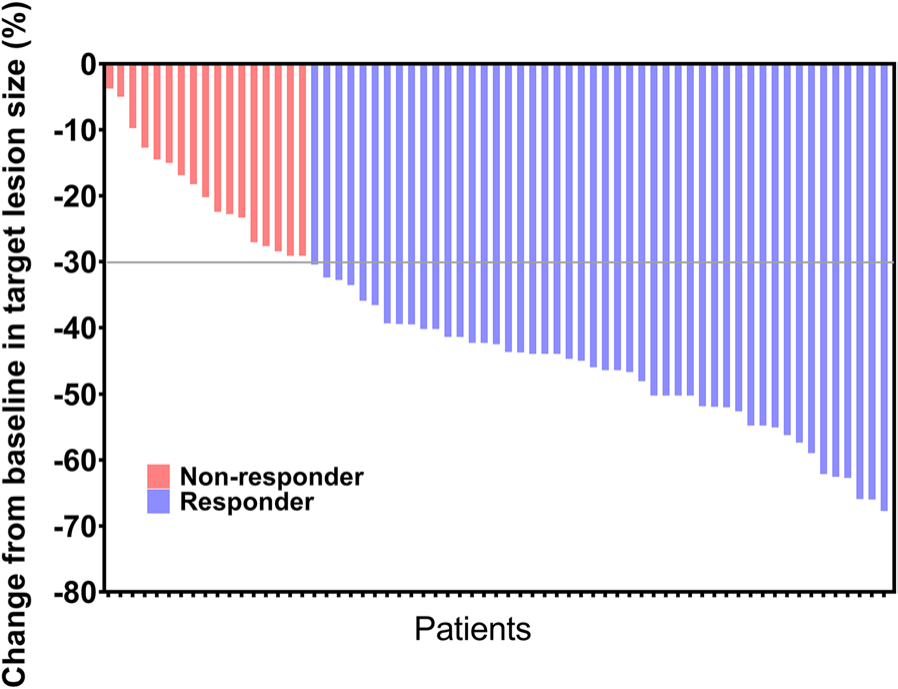
Change in target lesion size from baseline after SBRT

Baseline characteristics of all 66 patients with lung metastases of NSCLC are presented in Table 1. There were 45 (68%) males and 21 (32%) females; 36 (55%) squamous cell carcinomas (SCCs) and 30 (45%) adenocarcinomas (ADs); 28 (42%) patients with lung metastases ≤ 3 cm and 38 (58%) patients with lung metastases > 3 cm. As shown in Fig. 1, 49 (74.2%) of the 66 patients with lung metastases undergoing SBRT experienced PR (responders), while 17 (25.8%) experienced SD (non-responders) 1 month after SBRT. The individual tumor changes in target lesion size from baseline are shown in Fig. 2. The absolute numbers of memory CD8+ T, naive CD8+ T, memory CD4+ T and naïve CD4+ T cells were (0.18 ± 0.15) × 10^9^/L, (0.19 ± 0.13) × 10^9^/L, (0.41 ± 0.25) × 10^9^/L, and (0.13 ± 0.10) × 10^9^/L.

**Table 1:** Baseline characteristics of 66 NSCLC patients with lung metastases

Characteristic	N (%)
Age (years)	
≥ 60/< 60	44 (67%)/22 (33%)
Sex	
Male/female	45 (68%)/21 (32%)
Smoking history	
Never smoker/former smoker/current smoker	26 (39%)/4 (6%)/36 (55%)
Performance status	
0/1	31 (47%)/35 (53%)
Histological types	
SCC/AD	36 (55%)/30 (45%)
Metastatic status	
Isolated lung metastasis/multiple metastasis	50 (76%)/16 (24%)
Size of targeted lung metastases	
≤ 3 cm/> 3 cm	28 (42%)/38 (58%)
Primary T stage	
T1/T2/T3/T4	18 (27%)/29 (44%)/9 (14%)/10 (15%)
Primary N stage	
N0/N1/N2/N3	19 (29%)/20 (30%)/18 (27%)/9 (14%)
Primary AJCC stage	
I/II/III	13 (20%)/18 (27%)/35 (53%)

### Differences of immune factors between responders and non-responders

**Fig. 3:**
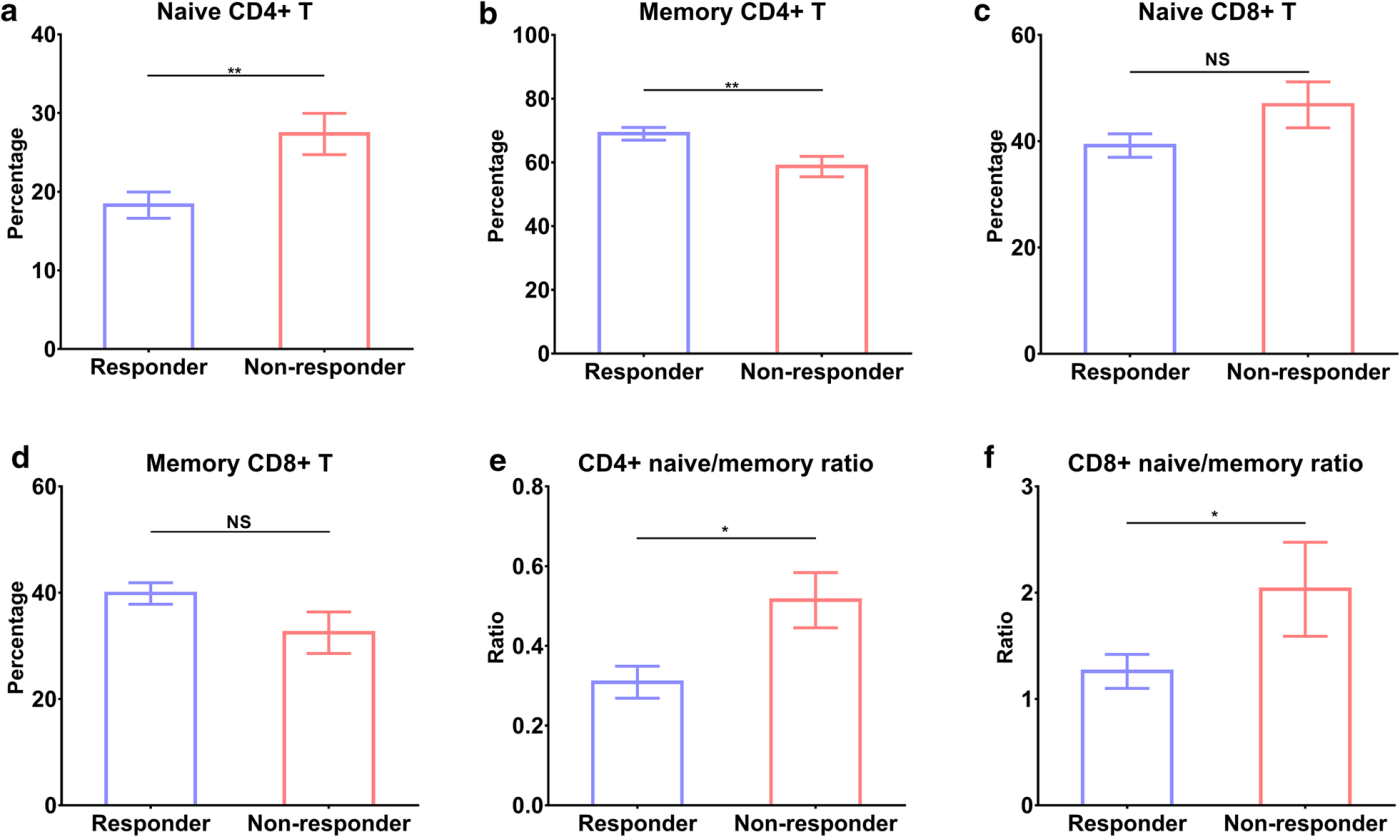
Differences in immune cells between responders and non-responders. **a** naïve CD4+ T, **b** memory CD4+ T, **c** naive CD8+ T, **d** memory CD8+ T, **e** CD4+ naïve/memory ratio, and **f** CD8+ naïve/memory ratio

As shown in Fig. 3, there were lower levels of naïve CD4+ T (18.29 ± 1.66 vs. 27.33 ± 2.623, *P* < 0.01), CD4+ naïve/memory ratio (0.31 ± 0.04 vs. 0.51 ± 0.07, *P* < 0.01), CD8+ naïve/memory ratio (1.26 ± 0.16 vs. 2.03 ± 0.44, *P* < 0.05), and higher memory CD4+ T (68.99 ± 1.99 vs. 58.71 ± 3.21, *P* < 0.05) and memory CD8+ T (39.86 ± 2.02 vs. 32.48 ± 3.90, *P* = 0.07) in responders compared to non-responders.

**Fig. 4:**
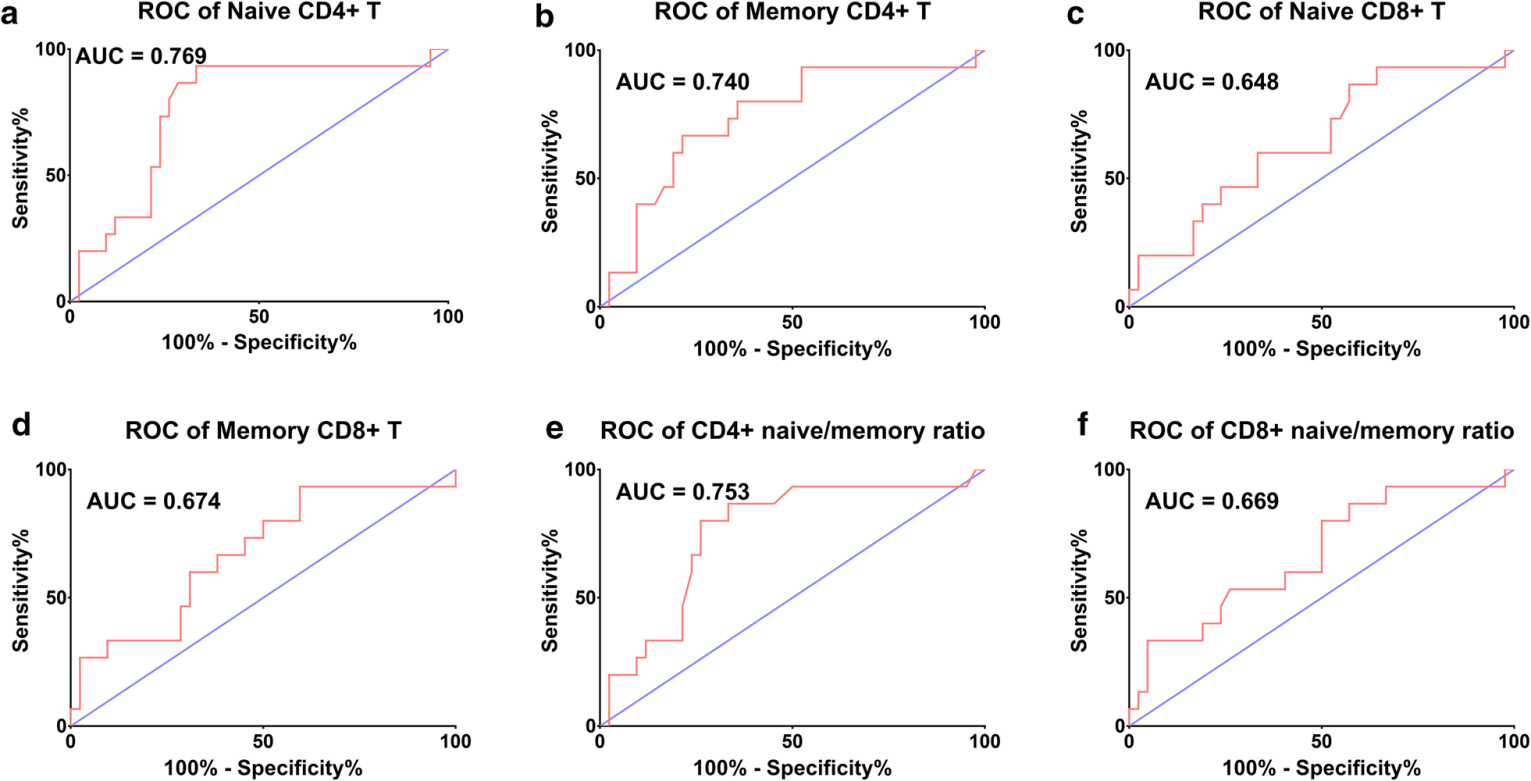
ROC curves of immune cells’ ability to discriminate between responders and non-responders for SBRT. **a** naïve CD4+ T, **b** memory CD4+ T, **c** naive CD8+ T, **d** memory CD8+ T, **e** CD4+ naïve/memory ratio, and **f** CD8+ naïve/memory ratio

ROC curves for immune factors’ ability to discriminate between responders and non-responders are shown in Fig. 4. The most sensitive and specific marker was naïve CD4+ T with an area under curve (AUC) of 0.769, whereas, memory CD4+ T with an AUC of 0.740, naïve CD8+ T with an AUC of 0.648, memory CD8+ T with an AUC of 0.674, CD4+ naïve/memory ratio with an AUC of 0.753, and CD8+ naïve/memory ratio with an AUC of 0.669 were somewhat less sensitive and specific.

**Fig. 5:**
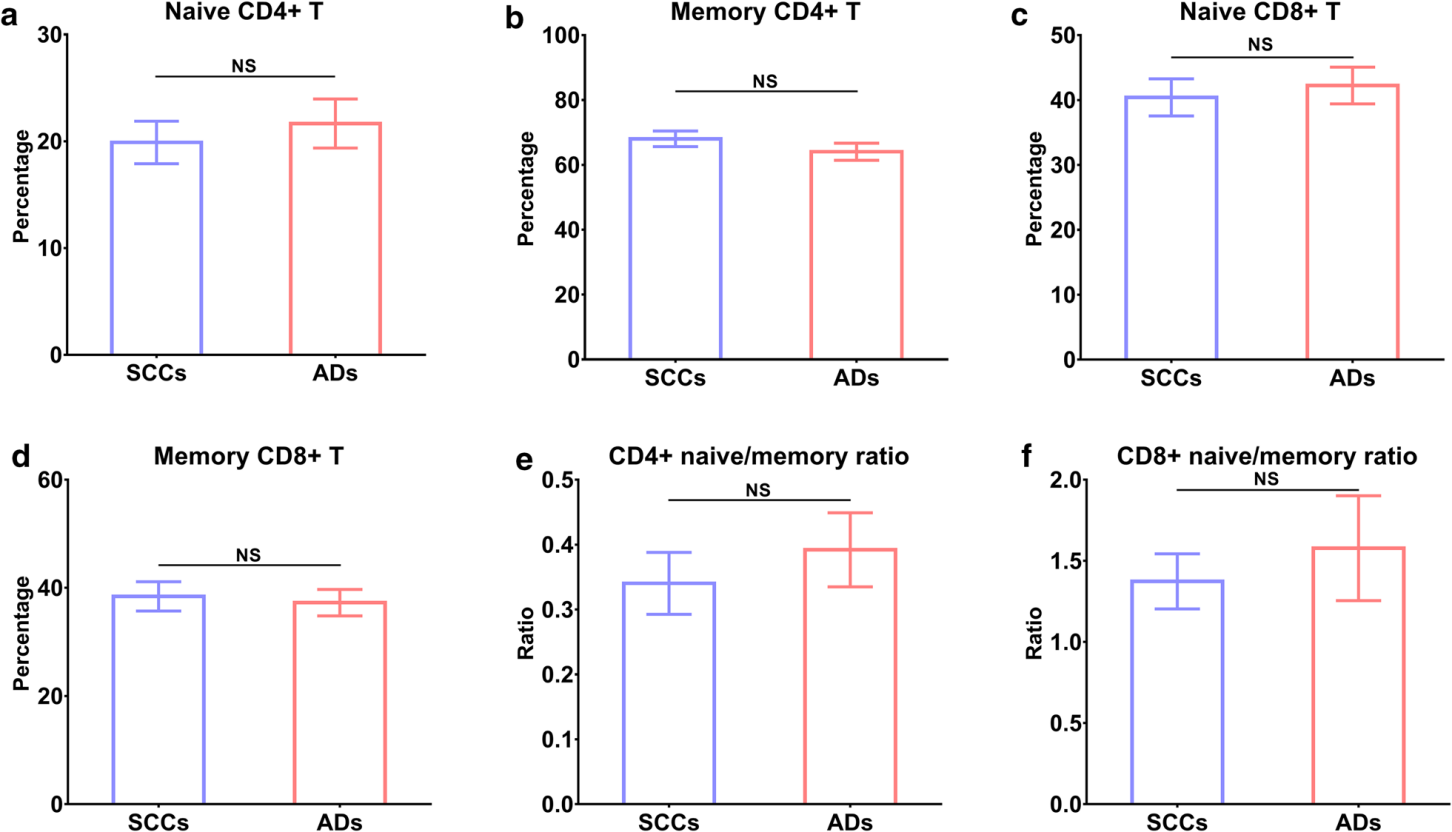
Differences in immune cells between SCCs and ADs. **a** naïve CD4+ T, **b** memory CD4+ T, **c** naive CD8+ T, **d** memory CD8+ T, **e** CD4+ naïve/memory ratio, and **f** CD8+ naïve/memory ratio

**Fig. 6:**
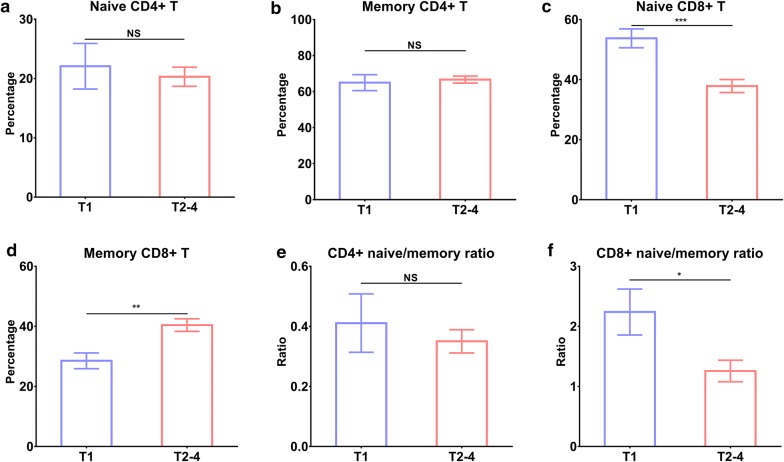
Differences in immune cells between patients with stage T1 and T2–4. **a** naïve CD4+ T, **b** memory CD4+ T, **c** naive CD8+ T, **d** memory CD8+ T, **e** CD4+ naïve/memory ratio, and **f** CD8+ naïve/memory ratio

We also compared immune cells between 36 SCCs and 30 ADs and found no significant difference between them (all *P* > 0.05, Fig. 5). Patients with stage T1 had higher levels of naïve CD8+ T (*P* < 0.001), CD8+ naïve/memory ratio (*P* < 0.05), and lower memory CD8+ T (*P* < 0.01) when compared to those with stage T2–4 (Fig. 6).

### Univariate logistic regression analysis

As shown in Table 2, low levels of naïve CD4+ T (OR: 16.25, 95% CI 3.17–83.13, *P* = 0.001), CD4+ naive/memory ratio (OR: 11.27, 95% CI 2.67–47.58, *P* = 0.001), and high levels of memory CD4+ T (OR: 0.14, 95% CI 0.04–0.50, *P* = 0.003) and memory CD8+ T (OR: 0.11, 95% CI 0.01–0.87, *P* = 0.037) predicted better tumor response to SBRT. The correlation between levels of naive CD8+ T, CD8+ naive/memory ratio, and BED_10_ and tumor response demonstrated strong trends (*P* = 0.054, 0.053, and 0.055, respectively).

**Table 2:** Univariate logistic regression analysis of predictors for tumor response

Predictors	OR (95% CI)	P
Age (years)		
≥ 60 vs. < 60	1.28 (0.38–4.22)	0.691
Sex		
Male vs. female	0.81 (0.25–2.59)	0.721
Smoking history		
Smoker vs. never smoker	0.47 (0.15–1.45)	0.189
Performance status		
1 vs. 0	0.72 (0.24–2.19)	0.568
Histological types		
AD vs. SCC	1.50 (0.49–4.54)	0.437
Metastatic status		
Multiple vs. isolated	2.13 (0.63–7.16)	0.223
Size of targeted lung metastases		
> 3 cm vs. ≤ 3 cm	1.49 (0.48–4.68)	0.491
Primary T stage		
T2–4 vs. T1	0.87 (0.26–2.94)	0.818
Primary N stage		
N1–3 vs. N0	1.43 (0.40–5.13)	0.580
Primary AJCC stage		
III vs. I–II	2.17 (0.43–10.98)	0.349
BED_10_		
High vs. low	0.31 (0.09–1.02)	0.055
Naive CD4+ T		
High vs. low	16.25 (3.17–83.13)	0.001
Memory CD4+ T		
High vs. low	0.14 (0.04–0.50)	0.003
Naive CD8+ T		
High vs. low	4.87 (0.97–24.37)	0.054
Memory CD8+ T		
High vs. low	0.11 (0.01–0.87)	0.037
CD4+ naive/memory ratio		
High vs. low	11.27 (2.67–47.58)	0.001
CD8+ naive/memory ratio		
High vs. low	4.00 (0.98–16.26)	0.053

### Multivariate logistic regression analysis

Univariate analytical findings with *P* < 0.1, including naive CD4+ T, memory CD4+ T, naive CD8+ T, memory CD8+ T, CD4+ naïve/memory ratio, CD8+ naïve/memory ratio, and BED_10_, were enrolled in multivariate analysis. Naïve CD4+ T (OR: 12.67, 95% CI 2.26–71.18, *P* = 0.004), memory CD4+ T (OR: 0.17, 95% CI 0.05–0.66, *P* = 0.010), memory CD8+ T (OR: 0.11, 95% CI 0.01–0.97, *P* = 0.047), and CD4+ naïve/memory ratio (OR: 8.50, 95% CI 1.90–38.14, *P* = 0.005) were independent predictors of tumor response to SBRT (Table 3).

**Table 3:** Multivariate logistic regression analysis of predictors for tumor response

Predictors	OR (95% CI)	P
Naive CD4+ T		
High vs. low	12.67 (2.26–71.18)	0.004
Memory CD4+ T		
High vs. low	0.17 (0.05–0.66)	0.010
Naive CD8+ T		
High vs. low	4.00 (0.76–20.91)	0.101
Memory CD8+ T		
High vs. low	0.11 (0.01–0.97)	0.047
CD4+ naive/memory ratio		
High vs. low	8.50 (1.90–8.14)	0.005
CD8+ naive/memory ratio		
High vs. low	2.98 (0.69–12.83)	0.143

## Discussion

Few studies have investigated tumor response predictors after SBRT. One of those, a recent study, revealed that the mean and maximum values of pre-SBRT standard uptake value could predict a complete response in lung metastases from various primary tumors 6 months after SBRT [27]. In addition, a minimum 20% shrinkage in lung lesion during the final SBRT was revealed to correlate positively with a complete response, 6 months after SBRT [27, 28]. Here, we have provided additional information, that peripheral memory CD4+ T, memory CD8+ T, naïve CD4+ T, and CD4+ naïve/memory ratio were independent tumor response predictors to SBRT in NSCLC lung metastases.

CD45RO has been identified as a common marker of all subdivisions of memory T-cells, such as subdivisions of the bone marrow and secondary lymphoid organs, and subdivisions of circulating and tissue-resident nature, but it is not known to mark T memory-stem cells [29]. The elimination of an antigen necessitates the generation of Memory T cells during cell-mediated immune responses, and these generated cells last months and years after the antigens are gone, causing quicker and bigger responses to secondary and ensuing antigen exposures [30]. Upon tumor antigen stimulation, activated memory CD4+ T cells respond very early to impede extensive replication or any significant impairment, either directly attacking the invading organism or providing assistance to B or cytotoxic T cells [31]. Memory CD8+ T cells have the ability to persist for years and kill tumor and virally infected cells [32].

The prognostic value of tumor-infiltrating memory T cells has been assessed by many researchers in lung cancer. Memory T cells that infiltrate tumors in lymph-node metastases reportedly are positive independent factors of prognosis for survival in patients with NSCLC [33]. A positive correlation between tumor-associated memory T cells and survival of SCLC patients has been shown to exist [34]. Interestingly, the correlation between memory T cells that infiltrate renal cell carcinoma and survival was negative, possibly due to impaired infiltrating lymphocytes within the renal cell carcinoma [35]. In our study, we found more memory CD4+ T and CD8+ T cells in responders than non-responders in lung metastases undergoing SBRT. In multivariate logistic regression analysis, memory CD4+ T and CD8+ T were independent predictors of tumor response to SBRT, consistent with the function of memory cells.

Also, we found fewer naïve CD4+ T cells, CD4+ naïve/memory ratio, and CD8+ naïve/memory ratio in responders than non-responders and an unfavorable predictive value of naive CD4+ T and CD4+ naïve/memory ratio for tumor response after SBRT. Recently, Su et al. [36] reported that the chemotaxis of circulating naive CD4+ T cells differentiating into Tregs in situ and causing immunosuppression of tumors trigger the infiltration of breast tumors by Tregs, which may explain our findings on naïve CD4+ T.

Several limitations exist in our study. First, different histological types were enrolled, including adenocarcinoma and squamous cell carcinoma. Second, the sample size of 66 patients was limited and the study contained an unavoidable selection bias. Third, different doses of SBRT were used in our study. Finally, although the tumor response was evaluated 1 month after SBRT, we were unable to evaluate it after 6 months, since most patients went back to their local hospitals 1 month after SBRT. Nevertheless, our study could potentially be a step towards providing additional biomarkers for predicting tumor response after SBRT.

## Conclusions

We revealed that peripheral memory CD4+ T, memory CD8+ T, naïve CD4+ T, and CD4+ naïve/memory ratio were independent predictors for tumor response to SBRT in NSCLC lung metastases. Larger, in-depth studies are necessary to verify our findings.

## Additional file

**Additional file 1: Figure S1.** Representative flow cytometry plots and gating for (A) memory CD4+ T and naïve CD4+ T cells, (B) memory CD8+ T and naive CD8+ T cells.

## Data Availability

All data included in our study are shown in our manuscript.

## Abbreviations

SBRT: stereotactic body radiotherapy
NSCLC: non-small cell lung cancer
AJCC: American Joint Committee on Cancer
PD: progressive disease
PR: partial response
SD: stable disease
CR: complete response
AD: adenocarcinoma
SCC: squamous cell carcinomas
