# Supplementary material for: Peripheral memory and naïve T cells in non-small cell lung cancer patients with lung metastases undergoing stereotactic body radiotherapy: predictors of early tumor response
Source: Cancer Cell Int. 2019 May 7;19:121. doi: 10.1186/s12935-019-0839-5 (PMC6505218; doi:10.1186/s12935-019-0839-5)
Supplement: Supplementary file 1 — Additional file 1: Figure S1. Representative flow cytometry plots and gating for (A) memory CD4+ T and naïve CD4+ T cells, (B) memory CD8+ T and naive CD8+ T cells. [file 12935_2019_839_MOESM1_ESM.docx]

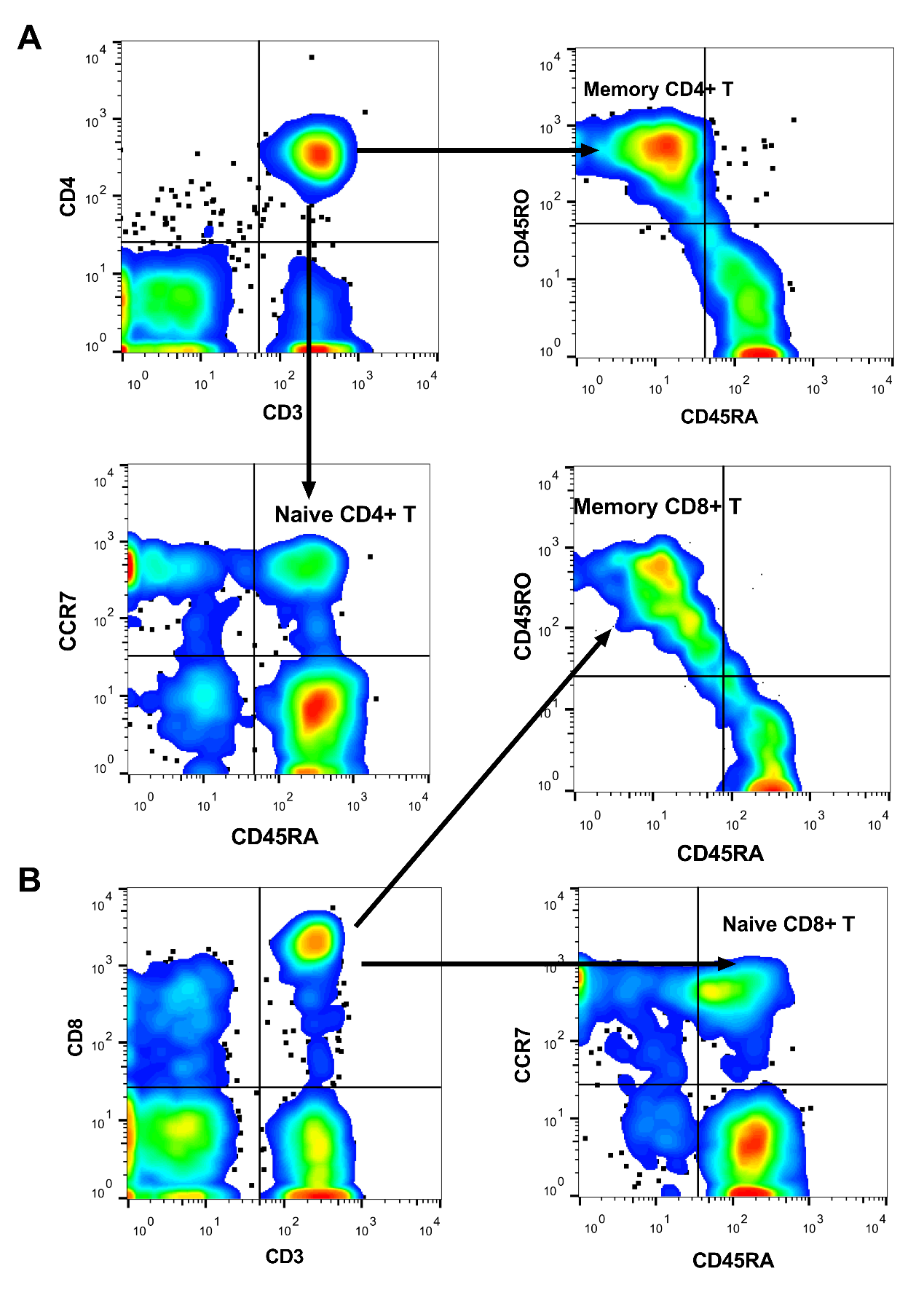


**Figure S1** Representative flow cytometry plots and gating for (A) memory CD4+ T and naïve CD4+ T cells, (B) memory CD8+ T and naive CD8+ T cells.
